# On Maximum Entropy Density Estimation with Relaxed Moment Constraints

**DOI:** 10.3390/e28030282

**Published:** 2026-03-02

**Authors:** Thi Lich Nghiem, Pierre Maréchal

**Affiliations:** 1 Informatics Department, Thuongmai University, 79 Ho Tung Mau Street, Cau Giay District, Hanoi 100000, Vietnam; lichnt72@tmu.edu.vn; 2 Institut de Mathématiques de Toulouse, Université de Toulouse, 118 Route de Narbonne, 31062 Toulouse, Cedex 9, France; 3 Équipe OPTIM-Optimisation, École Nationale de l’Aviation Civile, 7 Avenue Édouard Belin, 31055 Toulouse, Cedex 4, France

**Keywords:** maximum entropy, method of moments, species distribution, fenchel duality, relaxation

## Abstract

We study Maximum Entropy density estimation on continuous domains under finitely many moment constraints, formulated as the minimization of the Kullback–Leibler divergence with respect to a reference measure. To model uncertainty in empirical moments, constraints are relaxed through convex penalty functions, leading to an infinite-dimensional convex optimization problem over probability densities. The main contribution of this work is a rigorous convex-analytic treatment of such relaxed Maximum Entropy problems in a functional setting, without discretization or smoothness assumptions on the density. Using convex integral functionals and an extension of Fenchel duality, we show that, under mild and explicit qualification conditions, the infinite-dimensional primal problem admits a dual formulation involving only finitely many variables. This reduction can be interpreted as a continuous-domain instance of partially finite convex programming. The resulting dual problem yields explicit primal–dual optimality conditions and characterizes Maximum Entropy solutions in exponential form. The proposed framework unifies exact and relaxed moment constraints, including box and quadratic relaxations, within a single variational formulation, and provides a mathematically sound foundation for relaxed Maximum Entropy methods previously studied mainly in finite or discrete settings. A brief numerical illustration demonstrates the practical tractability of the approach.

## 1. Introduction

The Maximum Entropy principle provides a classical framework for estimating probability distributions from partial information, typically in the form of moment constraints. In its standard formulation, the problem is posed on a finite or discrete sample space and reduces to a finite-dimensional convex optimization problem, which has been extensively studied in statistics and machine learning.

In many situations of interest, however, the underlying sample space is continuous and no natural discretization is available. Estimating a probability density on a continuous domain under moment constraints then leads to an infinite-dimensional optimization problem, for which the usual finite-dimensional duality arguments no longer apply directly.

Such continuous-domain formulations arise naturally in a variety of applications, including density estimation for physical or environmental variables (e.g., wind or ocean directions, spatial intensity fields), inverse problems and signal processing, and nonparametric statistical modeling where the unknown distribution is supported on a continuum. In directional and circular statistics, for instance, probability densities on the unit circle or sphere are routinely modeled without discretization [1,2]. In these settings, discretization may introduce bias or artificial constraints, whereas a genuine functional formulation is often preferable.

Continuous or nonparametric variants of the Maximum Entropy principle have also been studied in the literature, with foundational contributions by Csiszár on *I*-divergence geometry and entropy-based inference under exact linear constraints [3,4]. While these works exploit convexity and information-theoretic inequalities to enforce strict moment conditions in a rigorous manner, other continuous-domain treatments proceed in a more formal manner. In many such approaches, the entropy functional and the moment constraints are written in a continuous setting and optimal densities are derived by variational arguments or by analogy with the discrete case. Although these derivations often lead to plausible expressions, the precise functional-analytic framework and the underlying duality structure are frequently left implicit or treated only at a formal level.

Classical treatments of the Maximum Entropy principle in information theory and statistical physics rely on the formal Lagrange multiplier arguments and variational calculus [5,6,7]. In practice, continuous problems are often handled either through discretization or binning strategies that yield finite-dimensional approximations, or via parametric exponential-family models. While frequently effective, such approaches depend on approximation choices that may influence stability and accuracy.

From a convex-analytic perspective, entropy minimization can be formulated within the theory of convex integral functionals [8]. In the discrete setting, Dudík et al. [9] developed a systematic framework for Maximum Entropy estimation with relaxed moment constraints. The present work extends this line of research to genuinely continuous domains, where the primal variable is an infinite-dimensional density.

The purpose of this paper is to provide a fully rigorous formulation of the continuous-domain Maximum Entropy problem. We consider the minimization of the Kullback–Leibler divergence with respect to a reference measure under finitely many (possibly relaxed) moment constraints, where the optimization variable is a probability density defined on a continuous domain. The primary problem is genuinely infinite-dimensional and is formulated in a space of measurable functions defined by integrability with respect to a prescribed family of feature functions.

More precisely, the functional framework is built upon a dual pair of Köthe-type spaces associated with the chosen feature family, allowing moment constraints to be well defined. This setting accommodates a wide range of feature functions, including unbounded ones, and provides a natural domain for entropy optimization.

Our main contribution is to show that, under explicit and mild assumptions ensuring well-posedness of the moment operators and nonemptiness of the relevant effective domains, this infinite-dimensional convex problem admits a dual formulation involving only finitely many variables. The analysis relies on convex integral functionals and an extension of Fenchel duality, and yields precise primal–dual optimality conditions.

The proposed framework is closely related in spirit to the discrete relaxed Maximum Entropy approach of Dudík et al. [9]. It can be viewed as its continuous-domain counterpart, in which the finite-dimensional structure of the dual problem is preserved while the primal variable becomes an infinite-dimensional density.

Since empirical moments are computed from finite samples, they are subject to sampling variability and measurement noise. Enforcing them as exact constraints may therefore lead to overfitting or even infeasibility. This observation motivates the introduction of relaxed constraints through convex penalty functions, which provide a principled way to incorporate uncertainty while retaining the variational structure of the Maximum Entropy principle.

The paper is organized as follows. Section 2 formulates the continuous Maximum Entropy problem and introduces relaxed moment constraints. Section 3 derives the dual problem and establishes primal–dual optimality conditions. Section 4 discusses several choices of relaxation potentials and their consequences for the dual formulation. A brief numerical illustration is presented in Section 5, and concluding remarks are provided in Section 6.

## 2. Statement of the Problem

We consider the optimization of the so-called *differential entropy* or its *relative* counterpart, the Kullback–Leibler divergence between probability measures.

Let $\Omega \subset R^{d}$ be a bounded domain. We assume throughout that Ω is endowed with the σ-algebra of Borel sets. The space of Borelian functions on Ω is denoted by Bor(Ω).

Points $x_{1},\ldots ,x_{n}$ are observed in Ω. These points are assumed to be drawn according to some unknown probability *P* on Ω, and the fundamental problem is that of recovering *P*. Feature functions $f_{1},\ldots ,f_{m}:\;\Omega \to R$ are chosen, from which the following statistics are derived:

(1) $$\langle f_{k}\rangle =\frac{1}{n}\sum_{j=1}^{n}f_{k}\left(x_{j}\right),\;k=1,\ldots ,m.$$

The functions $f_{1},\ldots ,f_{m}$, also called moment functions, define the moment constraints of the problem.

Recall that the above equation expresses the expectation of $f_{k}$ under the *empirical measure*

$$P_{\mathrm{emp}}=\frac{1}{n}\sum_{j=1}^{n}\delta_{x_{j}},$$

in which $\delta_{x}$ denotes the Dirac measure at x. The Maximum Entropy principle states, in its native form, that among all probability measures *P* that are consistent with the moment constraints

(2) $$\langle f_{k}\rangle =\int f_{k}\;dP,\;\;k=1,\ldots ,m,$$

one should select the probability $\bar{P}$, which minimizes the Kullback–Leibler relative entropy $K(P∥P_{\circ })$ of *P* with respect to $P_{\circ }$. Here, $P_{\circ }$ is a *reference measure*, supported in Ω, which encodes the prior knowledge one may have about *P*. If no prior knowledge is available, it is customary to choose $P_{\circ }$ to be the uniform measure on Ω. Recall that the Kullback–Leibler relative entropy of *P* with respect to $P_{\circ }$ is defined as

$$K\left(P∥P_{\circ }\right):=\left\{\begin{array}{cc} \int \ln\frac{dP}{dP_{\circ }}\;dP & \mathrm{if}\;P≺\;\;≺P_{\circ },\\ \infty & \mathrm{otherwise}. \end{array}\right.$$

Here, $dP/dP_{\circ }$ denotes the Radon–Nikodym derivative of *P* with respect to $P_{\circ }$, and the notation $P≺\;\;≺P_{\circ }$ means that *P* is *absolutely continuous* with respect to $P_{\circ }$. We are facing the following optimization problem:

$$\left(P_{\circ }\right)\;\left|\;\begin{array}{ccc} \mathrm{Minimize} & & K(P∥P_{\circ })\\ s.t. & & 1=\int_{\Omega }dP,\;y=\int_{\Omega }f\;dP. \end{array}\right.$$

The shorthand *s.t.* stands for *subject to*, while $y=\left(y_{1},\ldots ,y_{m}\right)\in R^{m}$ is the vector whose *k*-th component equals $\langle f_{k}\rangle$ and f is the $R^{m}$-valued function whose *k*-th component equals $f_{k}$.

Problem $\left(P_{\circ }\right)$ is an infinite-dimensional convex problem with finitely many linear constraints. Although the optimized variable *P* lies in the space of Radon measures on Ω, it is equivalent to searching for a density with respect to $P_{\circ }$. As a matter of fact, minimizing the Kullback–Leibler divergence discards measures that are not absolutely continuous with respect to $P_{\circ }$.

Since empirical moments are computed from finite samples, they are subject to sampling variability and measurement noise. Enforcing them as exact constraints may therefore lead to overfitting or infeasible formulations; this motivates the introduction of relaxed constraints. Following Ref. [9], we introduce, for the purpose of relaxation, a convex *potential function* $U:R^{m}\to \left(-\infty ,\infty \right]$.

We therefore obtain the relaxed, functional counterpart of Problem $\left(P_{\circ }\right)$, namely

$$\left(P\right)\;\left|\;\begin{array}{ccc} \mathrm{Minimize} & & H(p)+U(Fp)\\ s.t. & & 1=Ip, \end{array}\right.$$

in which the variable *p* is the Radon–Nikodym derivative of the searched measure *P* with respect to $P_{\circ }$. The *neg-entropy* functional *H* is defined by

$$H\left(p\right):=\int h_{\circ }\left(p(x)\right)\;dP_{\circ }\left(x\right)\;\mathrm{with}\;h_{\circ }\left(t\right):=\left\{\begin{array}{ccc} t\ln t & \mathrm{if} & t>0,\\ 0 & \mathrm{if} & t=0,\\ \infty & \mathrm{if} & t<0 \end{array}\right.$$

and the linear operators F and I are, respectively, defined by

$$Fp=\int_{\Omega }f\left(x\right)\;p\left(x\right)\;dP_{\circ }\left(x\right)\;\mathrm{and}\;Ip=\int_{\Omega }p\left(x\right)\;dP_{\circ }\left(x\right).$$

For convenience, we group these linear operators into a single moment operator

$$A:p\mapsto \left(Ip,Fp\right)\in R\times R^{m},$$

which collects the normalization and moment constraints. This notation will be used throughout the sequel. The potential function *U* is generally a convex non-negative function, which attains its minimum value at y. The case of non-relaxed constraint [9] corresponds to the choice $U_{y}\left(s\right)=U_{y}^{\left(0\right)}\left(s\right)=\delta \left(s|\left\{y\right\}\right)$. Recall that the *indicator function* of a set *S* is the function

$$\delta \left(s|\;S\right):=\left\{\begin{array}{cc} 0 & \mathrm{if}\;s\in S,\\ \infty & \mathrm{otherwise}. \end{array}\right.$$

Clearly, this choice enforces the equality Ap=y. In [9], the case of box constraints was also considered. It corresponds to the choice $U_{y}\left(s\right)=U_{y}^{\left(1\right)}\left(s\right)=\delta \left(s|B_{y}\right)$, in which *B* is box

$$B_{y}=\prod_{k=1}^{m}\left[y_{k}-\beta_{k},y_{k}+\beta_{k}\right]$$

with $\beta =\left(\beta_{1},\ldots ,\beta_{m}\right)\in \left(R_{+}^{*}\right)$. In this finite-dimensional setting, the *convex conjugate* of $\delta \left(\cdot |B_{y}\right)$ coincides with the *support function* of the set $B_{y}$, namely

$$\delta {\left(\cdot |B_{y}\right)}^{★}\left(\sigma \right)=\underset{s\in B_{y}}{\sup}\langle \sigma ,s\rangle ,$$

where the pairing is the standard Euclidean inner product. Since the convex conjugate of $\delta \left(\cdot |B_{y}\right)$ fails to be differentiable, one may consider an approximation of the box constraint that yields a differentiable conjugate, as in Section 5.2 of [9]. This will also be considered in the present paper. The *least square* relaxation corresponds to the choice

$$U_{y}\left(s\right)=U_{y}^{\left(2\right)}\left(s\right)=\frac{1}{2\alpha }{∥\;y-s\;∥}^{2},$$

in which ∥·∥ denotes the Euclidean norm and α is some positive constant. We may also consider, more generally, the choice

$$U\left(s\right)=\frac{1}{2\alpha }{∥\;y-s\;∥}_{Q}^{2}$$

in which ${∥\;z\;∥}_{Q}^{2}={\langle z,z\rangle }_{Q}=\langle z,Qz\rangle$ where *Q* is a positive definite symmetric matrix. An interesting example of this is obtained if we let Q=Σ, with Σ an estimate of the covariance matrix of the vector y: the relaxation then accounts for the variance of each *principal component* of the data vector regarded as a random vector.

Implicitly, the above problem is posed in a space of measurable functions for which all moment constraints are well defined. More precisely, the natural domain of the optimization problem consists of functions that are integrable against the chosen family of feature (moment) functions.

This space is a subspace of $L_{P_{\circ }}^{1}\left(\Omega \right)$, the space of measurable functions that are integrable with respect to the reference measure $P_{\circ }$, and coincides with $L_{P_{\circ }}^{1}\left(\Omega \right)$ when all feature functions are bounded. In general, however, it is a proper subspace, since the moment operator is only well defined for densities *p* such that the pointwise products $pf_{k}$, defined by $\left(pf_{k}\right)\left(x\right)=p\left(x\right)f_{k}\left(x\right)$, belong to $L_{P_{\circ }}^{1}\left(\Omega \right)$ for every k∈{1,…,m}.

More generally, for a family of feature functions Φ, the natural workspace of the moment problem is the Köthe dual space

(3) $$L_{P_{\circ }}\left(\Omega ;\Phi \right):=\left\{\;p\in \mathrm{Bor}\left(\Omega \right)\;|\;\forall f\in \Phi ,\;pf\in L_{P_{\circ }}^{1}\left(\Omega \right)\;\right\}.$$

In the next section, we shall explore the dualization of problem (P) and show how this dualization provides algorithms for effective computation of Maximum Entropy solutions in our infinite-dimensional setting, without having to discretize the statistical problem under consideration.

## 3. Maximum Entropy Solutions

This section derives the dual formulation of the relaxed Maximum Entropy problem. Although the primary variable is an infinite-dimensional density, convex duality allows the problem to be reduced to the optimization of finitely many dual variables (Lagrange multipliers). The analysis relies on standard tools from convex analysis—in particular, convex conjugates, subdifferentials, and Fenchel duality—which provide a natural framework for establishing duality and optimality conditions. Standard references include [10,11,12], while results on convex integral functionals can be found in [8,13,14]. A recent overview in the context of entropy optimization is provided in [15].

For convenience, we define on $R\times R^{m}$ the concave function

$$G\left(\eta_{\circ },\eta \right):=-\delta \left(\eta_{\circ }|\left\{1\right\}\right)-U\left(\eta \right)$$

and rewrite (P) in the equivalent form

$$\left(P\right)\;\left|\;\begin{array}{ccc} \mathrm{Minimize} & & \left(H-G\circ A)(p\right)\\ s.t. & & p\in L_{P_{\circ }}\left(\Omega ;\Phi \right) \end{array}\right.$$

in which Ap:=(Ip,Fp). Here, G∘A denotes the composition of functions, that is, (G∘A)(p)=G(Ap). We shall also write U(η)=V(y−η) with *V* a proper convex lower semi-continuous (and most often even) function. We note that the dual problem associated with (P) reads

$$\left(D\right)\;\left|\;\begin{array}{ccc} \mathrm{Maximize} & & D\left(\lambda_{\circ },\lambda \right):=\left(G_{★}-H^{★}\circ A^{★}\right)\left(\lambda_{\circ },\lambda \right)\\ s.t. & & \left(\lambda_{\circ },\lambda \right)\in R\times R^{m}. \end{array}\right.$$

The symbol ^★^ can denote different, though related, mathematical notions depending on context. When used as a superscript on a function, as in $H^{★}$, it typically refers to the *convex conjugate* (or Legendre–Fenchel transform) of *H*. When used as a subscript, as in $G_{★}$, it denotes the *concave conjugate*. In the context of linear operators, the same symbol used as a superscript, as in $A^{★}$, denotes the *adjoint* of the operator A.

Let us clarify the notion of adjunction being referred to here: in our context, the operator A is well-defined from the space $L_{P_{\circ }}\left(\Omega ;\Phi \right)$ with $\Phi =\left\{1_{\Omega },f_{1},\ldots ,f_{m}\right\}$ to $R^{1+m}$, and its adjoint is defined with respect to the pairing of $L_{P_{\circ }}\left(\Omega ;\Phi \right)$ with

$$L_{P_{\circ }}^{★}\left(\Omega ;\Phi \right):=\left\{\;\varphi \in \mathrm{Bor}\left(\Omega \right)\;|\;\forall p\in L_{P_{\circ }}\left(\Omega ;\Phi \right),\;p\varphi \in L_{P_{\circ }}^{1}\left(\Omega \right)\;\right\}$$

realized via the standard integral bilinear product

(4) $$\langle p,\phi \rangle :=\int_{\Omega }p\left(x\right)\phi \left(x\right)\;dP_{\circ }\left(x\right).$$

An immediate computation shows that the adjoint of A is then given by

$$\begin{array}{cccc} A^{★}: & R^{1+m} & ⟶ & L_{P_{\circ }}^{★}\left(\Omega ;\Phi \right)\\ & \left(\lambda_{\circ },\lambda \right) & ⟼ & \lambda_{\circ }+\langle \lambda ,f\rangle . \end{array}$$

We use the notation ri(C) for the relative interior of a convex set *C*, that is, the interior taken with respect to the affine hull of *C* (see [10]).

**Theorem** **1.** *Assume That The Following Conditions Hold:*

(i) *for every k∈{1,…,m}, the function $f_{k}$ is integrable over every set of finite measure;*
(ii) *ri(AdomH)∩ri(domG)≠∅;*
(iii) *$\mathrm{ri}\mathrm{dom}G_{★}\cap \mathrm{ri}\mathrm{dom}\left(H^{★}\circ A^{★}\right)\neq \emptyset$;*
(iv) *the function V underlying the definition of G is closed and proper convex*.

*If $\left({\bar{\lambda }}_{\circ },\bar{\lambda }\right)$ maximizes the dual function D, then the density $\bar{p}$ defined on* Ω *by*

$$\bar{p}\left(x\right):={\left(h_{\circ }^{★}\right)}^{\prime }\left(\left[A^{★}\left({\bar{\lambda }}_{\circ },\bar{\lambda }\right)\right]\left(x\right)\right)=\exp\left({\bar{\lambda }}_{\circ }-1\right)\cdot \exp\langle \bar{\lambda },f\left(x\right)\rangle$$

*is optimal in problem (P).*

**Proof.**  *Step 1.* By Fenchel duality, thanks to Condition (ii), the optimal value v(P) of Problem (P) coincides with that of the dual problem (D), and dual attainment is achieved:

$$v\left(P\right)=\max\left\{\;D\left(\lambda_{\circ },\lambda \right)\;|\;\left(\lambda_{\circ },\lambda \right)\in R^{1+m}\;\right\}.$$

See, e.g., Theorem 4 of [15].

*Step 2.* We now turn to the computation of the dual function. We start by computing the concave conjugate of *G*:

$$\begin{array}{ccc} G_{★}\left(\lambda_{\circ },\lambda \right) & := & \inf\left\{\langle \left(\lambda_{\circ },\lambda \right),\left(\eta_{\circ },\eta \right)\rangle -G\left(\eta_{\circ },\eta \right)\;|\;\left(\eta_{\circ },\eta \right)\in R^{1+m}\;\right\}\\ & = & \inf\left\{\;\lambda_{\circ }\eta_{\circ }+\langle \lambda ,\eta \rangle +\delta \left(\eta_{\circ }|\left\{1\right\}\right)+U\left(\eta \right)\;|\;\left(\eta_{\circ },\eta \right)\in R^{1+m}\;\right\}\\ & = & \lambda_{\circ }+\inf\left\{\;\langle \lambda ,\eta \rangle +V\left(y-\eta \right)\;|\;\eta \in R^{m}\;\right\}\\ & = & \lambda_{\circ }-\sup\left\{\;-\langle \lambda ,\eta \rangle -V\left(y-\eta \right)\;|\;\eta \in R^{m}\;\right\}\\ & = & \lambda_{\circ }+\langle y,\lambda \rangle -V^{★}\left(\lambda \right), \end{array}$$

in which the last equality is obtained by the change of variable $\eta^{\prime }=y-\eta$. Let us now compute the convex conjugate of *H* in the pairing (4). Condition (i) implies, via Lemma 1 and Proposition 1 [16], that both $L_{P_{\circ }}\left(\Omega ;\Phi \right)$ and $L_{P_{\circ }}^{★}\left(\Omega ;\Phi \right)$ are decomposable. Moreover, the entropy kernel $h_{\circ }\left(t\right)$ is a measurable integrand that is proper convex and lower semi-continuous. Its convex conjugate is provided by

$$h_{\circ }^{★}\left(\tau \right)=\exp\left(\tau -1\right),\;\tau \in R.$$

Since $L_{P_{\circ }}\left(\Omega ;\Phi \right)$ is decomposable and *H* has a nonempty effective domain, it follows that, for every $\varphi \in L_{P_{\circ }}^{★}\left(\Omega ;\Phi \right)$,

(5) $$H^{★}\left(\varphi \right)=\int_{\Omega }\exp\left(\varphi (x)-1\right)\;dP_{\circ }\left(x\right).$$

Otherwise expressed, conjugacy through the integral sign holds. Now, $h_{\circ }^{★★}=h_{\circ }$ since $h_{\circ }$ is proper convex and lower semi-continuous. Moreover, it is readily seen that

$$L_{P_{\circ }}^{★★}\left(\Omega ;\Phi \right):=\left\{\;p\in \mathrm{Bor}\left(\Omega \right)\;|\;\forall \varphi \in L_{P_{\circ }}^{★}\left(\Omega ;\Phi \right),\;p\varphi \in L_{P_{\circ }}^{1}\left(\Omega \right)\;\right\}=L_{P_{\circ }}\left(\Omega ;\Phi \right).$$

See Property (f) of [16] (p. 207). It follows that conjugacy through the integral sign applies one more time: for all $p\in L_{P_{\circ }}\left(\Omega ;\Phi \right)$,

(6) $$H^{★★}\left(p\right)=\int_{\Omega }h_{\circ }^{★★}\left(p(x)\right)\;dP_{\circ }\left(x\right)=H\left(p\right).$$

*Step 3.* We shall prove here that the dual solution satisfies

(a) $\bar{p}\in \partial H^{★}\left(A^{★}\left({\bar{\lambda }}_{\circ },\bar{\lambda }\right)\right)$;
(b) $H^{★}\circ A^{★}$ has gradient $A\bar{p}$ at $\left({\bar{\lambda }}_{\circ },\bar{\lambda }\right)$.

The conclusion then follows from Theorem 6 of [15], since $H^{★★}=H$ (see Equation (6)) and $G_{★★}=G$ under Assumption (iv). Clearly, $h_{\circ }$ is differentiable throughout R, and its derivative equals itself. By integrating the subdifferential inequality as in Proposition 6 of [15], for example, we see that the obvious inclusion ${\left(h_{\circ }^{★}\right)}^{\prime }\left(\tau \right)\in \partial h_{\circ }^{★}\left(\tau \right)$ gives rise to the following inclusion:

$${\left(h_{\circ }^{★}\right)}^{\prime }\circ \varphi \in \partial H^{★}\left(\varphi \right)$$

for every $\varphi \in L_{P_{\circ }}^{★}\left(\Omega ;\Phi \right)$, thanks to Equation (5). Replacing φ by $A^{★}\left({\bar{\lambda }}_{\circ },\bar{\lambda }\right)$ yields (a). Finally, differentiating

$$\left(H^{★}\circ A^{★}\right)\left(\lambda_{\circ },\lambda \right)=\int_{\Omega }\exp\left(\lambda_{\circ }+\langle \lambda ,f(x)\rangle \right)dx$$

through the integral sign shows that

$$\nabla \left(H^{★}\circ A^{★}\right)\left({\bar{\lambda }}_{\circ },\bar{\lambda }\right)=A\bar{p}.$$

Thus, (b) is also satisfied, and the proof is complete. □

As a byproduct of the above theorem, we see that the dual problem corresponding to Problem (P) consists of maximizing the function

(7) $$D\left(\lambda_{\circ },\lambda \right):=\lambda_{\circ }+\langle y,\lambda \rangle -V^{★}\left(\lambda \right)-\exp\left(\lambda_{\circ }-1\right)\int_{\Omega }\exp\langle \lambda ,f(x)\rangle dP_{\circ }\left(x\right).$$

By the first-order optimality condition for concave maximization (often referred to as Fermat’s principle; see [10]), we find that $\left({\bar{\lambda }}_{\circ },\bar{\lambda }\right)$ is dual optimal if and only if

$$\left(0,0\right)\in \partial D\left({\bar{\lambda }}_{\circ },\bar{\lambda }\right).$$

Here, the subdifferential is taken in the sense of concavity. Recall that the dual function is concave; thus, sufficiency is granted along with necessity. It is readily checked that the latter condition is equivalent to

$$\exp\left({\bar{\lambda }}_{\circ }-1\right)={\left(\int_{\Omega }\exp\langle \bar{\lambda },f\left(x\right)\rangle \;dx\right)}^{-1}\;\mathrm{and}\;0\in \partial \tilde{D}\left(\bar{\lambda }\right),$$

in which

$$\tilde{D}\left(\lambda \right):=\langle \lambda ,y\rangle -V^{★}\left(\lambda \right)-\ln\int_{\Omega }\exp\langle \lambda ,f(x)\rangle \;dx.$$

Therefore, maximizing the dual function in (7) boils down to maximizing the function $\tilde{D}$. We observe that $\tilde{D}$ is concave and upper semi-continuous, as a consequence of the convexity and lower semi-continuity of the log-Laplace transform. We note that evaluating $\tilde{D}\left(\lambda \right)$ relies on some numerical integration in dimension *m*. This is by no means a limitation, due to the existence of powerful numerical integration algorithms such as Monte Carlo integration, for example.

Let us summarize the above achievements. If the assumptions of Theorem 1 are satisfied, which clearly depend on the choice of the potential *V* and on that of the feature functions $f_{1},\ldots ,f_{m}$, we compute the optimal vector $\bar{\lambda }$ by maximizing $\tilde{D}$, and we then obtain the Maximum Entropy solution $\bar{p}$ via the formula

$$\bar{p}\left(x\right)=\frac{\exp\langle \bar{\lambda },f\left(x\right)\rangle }{\int_{\Omega }\exp\langle \bar{\lambda },f\left(x\right)\rangle \;dx},\;x\in \Omega .$$

Some potentials *V* give rise to nice, differentiable, concave-unconstrained maximization problems. This happens whenever the conjugate $V^{★}$ has a full domain and is differentiable. A standard example is provided by the least square relaxation. However, in general, the function $V^{★}$ may suffer from drawbacks such as failing to have a full domain (so that the dual problem is constrained) or being non-differentiable. A standard example is provided by the case of box constraints.

In the smooth unconstrained case, quasi-Newtonian methods, such as the BFGS algorithm, are expected to provide a fast and accurate solution. If $V^{★}$ is not differentiable, the situation can still be addressed using the *proximal gradient algorithm*. As a matter of fact, in the latter case, the function to be maximized is then a *composite* concave function, that is to say, a sum of two concave functions, one being differentiable, the other being non-differentiable. The reader interested in the optimization of composite functions and the proximal gradient algorithm may refer to [17,18] and the references therein.

## 4. Relaxation

Following Ref. [9], we explore a number of potential functions to be used for the reconstruction of multivariate distributions. We assume throughout that the domain of $H^{★}\circ A^{★}$ is nonempty—a mild assumption on the family of feature functions.

**No relaxation at all.** In this case, $V\left(\eta \right)=\delta \left(\eta |\left\{0\right\}\right)$. The convex conjugate $V^{★}\left(\lambda \right)$ is then the function identically equal to zero. Condition (QC) reads y∈ri(FdomH) in this case, and Condition $\left(QC^{★}\right)$ is automatically satisfied. The reason is that the effective domain of $G_{★}$ is then the whole of $R^{m}$, and that $\mathrm{ri}\mathrm{dom}(H^{★}\circ A^{★})$ is nonempty, since the relative interior of a nonempty convex set is nonempty.

**Box constraints.** The function *V* is defined in this case by

$$V\left(\eta \right)=\delta \left(\eta |\prod_{k=1}^{n}\left[-\beta_{k},\beta_{k}\right]\right)=\sum_{k=1}^{n}\delta \left(\eta_{k}|\left[-\beta_{k},\beta_{k}\right]\right).$$

Its convex conjugate is then given by

$$V^{★}\left(\lambda \right)=\sum_{k=1}^{n}\beta_{k}\left|\;\lambda_{k}\;\right|={∥\;\beta ⊙\lambda |\;∥}_{1},$$

in which ⊙ denotes the pointwise product. In this case, Condition (QC) reads

$$\left(\prod_{k=1}^{m}\left(y_{k}-\beta_{k},y_{k}+\beta_{k}\right)\right)⋂\mathrm{ri}\left(F\mathrm{dom}H\right)\neq \emptyset .$$

Condition $\left(QC^{★}\right)$ is, again, automatically satisfied. Since the corresponding function $\tilde{D}$ is a composite function, the maximization of $\tilde{D}$ will be efficiently performed by the proximal gradient algorithm.

**Smoothed inner approximation of box constraints.** Let $\psi_{\beta }:R\to \left(-\infty ,\infty \right]$ be defined by

$$\psi_{\beta }\left(\eta \right)=\left\{\begin{array}{cc} \frac{\eta }{2}\ln\frac{\beta +\eta }{\beta -\eta }+\beta \ln\sqrt{1-\frac{\eta^{2}}{\beta^{2}}} & \mathrm{if}\;\eta \in (-\beta ,\beta ),\\ \infty & \mathrm{otherwise}. \end{array}\right.$$

**Figure 1:**
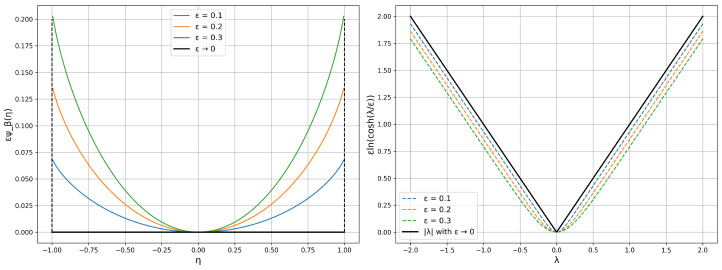
Plots of $\varepsilon \psi_{\beta }\left(\eta \right)$ and its conjugate for β=1 and various values of ε.

The smooth inner approximation of the box constraints with the same box as in the previous paragraph, together with its convex conjugate, are given, respectively, by

$$V\left(\eta \right)=\sum_{k=1}^{m}\varepsilon_{k}\psi_{\beta_{k}}\left(\eta_{k}\right)\;\mathrm{and}\;V^{★}\left(\lambda \right)=\sum_{k=1}^{m}\beta_{k}\varepsilon_{k}\ln\mathrm{ch}\left(\frac{\lambda_{k}}{\varepsilon_{k}}\right),$$

in which $\varepsilon_{k}$ controls the proximity of V(η) to the target box constraint potential and, thereby, the proximity of the corresponding conjugate functions. Figure 1 illustrates the approximation of the box constraint potential and its conjugate. We note that the constraint qualification condition and its dual counterpart are exactly as in the box constraint case. The advantage here is that the dual function is differentiable, allowing the use of powerful quasi-Newtonian optimization methods.

**Least squares.** Given a symmetric positive definite matrix *Q*, the weighted least squares relaxation corresponds to the case where

$$V\left(\eta \right)=\frac{1}{2\alpha }{∥\;\eta \;∥}_{Q^{-1}}^{2}\;\mathrm{and}\;V^{★}\left(\lambda \right)=\frac{\alpha }{2}{∥\;\eta \;∥}_{Q}^{2}.$$

The standard least squares relaxation is obtained by letting Q=I, the identity matrix. If an empirical covariance matrix Σ of the moment vector is available, one may choose Q=Σ. The relaxation term then involves the quadratic form $\eta^{⊤}\Sigma^{-1}\eta$, i.e., a Mahalanobis-type distance. In the eigenbasis of Σ, this amounts to weighting each principal component inversely to its variance, so that directions with larger uncertainty are penalized less strongly.

Remarkably, things once again look nicer on the dual side: no matrix inversion is required to evaluate $\tilde{D}\left(\lambda \right)$. Finally, the constraint qualification and its dual counterpart are automatically fulfilled here.

## 5. Experimental Illustration

This section provides a brief numerical validation of the proposed continuous-domain Maximum Entropy formulation. The goal is not to perform an extensive empirical benchmark, but to illustrate that the dual approach yields stable and meaningful density reconstructions without discretizing the domain. We consider both synthetic data and a real-world meteorological dataset.

### 5.1. Synthetic Angular Densities

We first consider synthetic directional data on the unit circle, a classical setting in circular statistics. Three target densities are used: a unimodal von Mises distribution, a symmetric bimodal mixture, and a trimodal mixture. For each case, n=2000 samples are generated.

Densities are reconstructed using trigonometric feature functions

$$\varphi_{k}\left(\theta \right)=\left(\cos(k\theta ),\sin\left(k\theta \right)\right),\;k=1,\ldots ,K,$$

with K∈{1,2,4,8}. A quadratic relaxation is employed, and the resulting finite-dimensional dual problem is solved using a quasi-Newton (BFGS) method. Normalization constants are evaluated by numerical quadrature.

**Figure 2:**
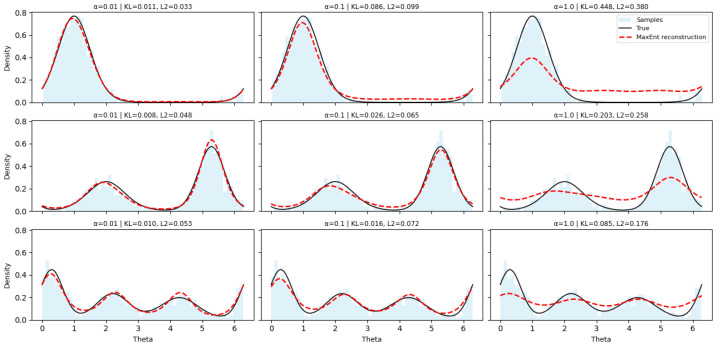
Reconstructed MaxEnt angular densities for unimodal, bimodal, and trimodal distributions under varying regularization strengths α∈{0.01,0.1,1.0}. Each subplot shows the true density (black solid), reconstructed density (red dashed), and sampled histogram (blue). Smaller α values preserve sharper peaks and fine directional structure, while larger α values yield smoother but less distinct reconstructions.

Representative reconstructions are shown in Figure 2. In all cases, the Maximum Entropy solution correctly captures the qualitative structure of the target distribution, including multimodality, without any discretization of the domain. The regularization parameter α controls smoothness: small values preserve sharp features, whereas larger values produce smoother densities.

### 5.2. Wind Direction Reconstruction

We next evaluate the method on a real meteorological dataset consisting of aircraft-based wind measurements over Europe (January 2018) [19]. The main recorded variables relevant to our study are summarized in Table 1.

The data include horizontal wind components $\left(w_{x},w_{y}\right)$, from which wind directions are computed as

$$\theta =\mathrm{mod}(\mathrm{atan}2\left(w_{y},w_{x}\right),2\pi ).$$

A random subset of $N=10^{4}$ observations is used.

As a reference, we compare the proposed Maximum Entropy estimator with a circular kernel density estimate (KDE). Reconstruction quality is assessed using the $L^{2}$ distance, the Kullback–Leibler divergence, and held-out log-likelihood.

**Figure 3:**
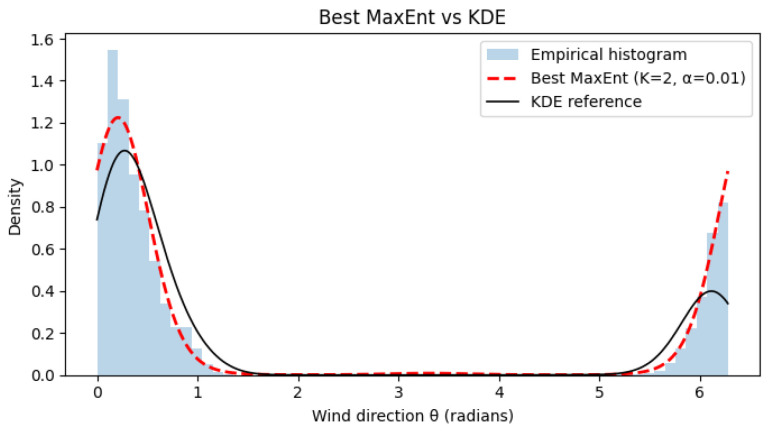
Reconstructed wind direction distribution using MaxEnt with K=2, $\alpha =10^{-2}$. The MaxEnt estimate (red dashed) accurately captures the two dominant wind regimes while maintaining smoothness and stability.

**Figure 4:**
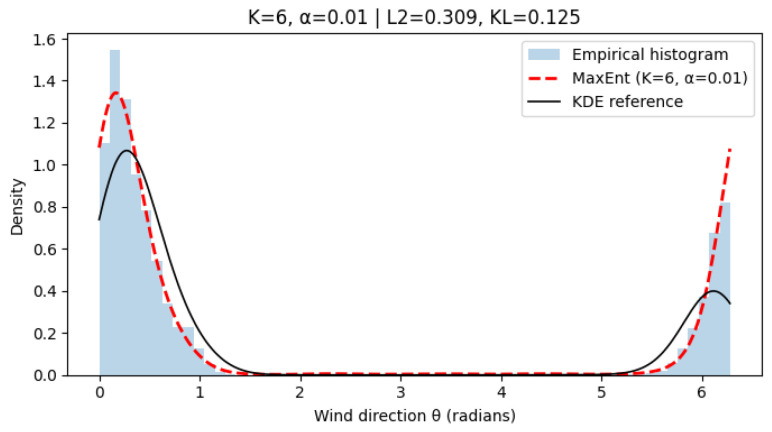
Reconstructed wind direction distribution using MaxEnt with K=6, $\alpha =10^{-2}$. The model achieves improved alignment with the KDE reference while preserving numerical stability and smooth transitions.

**Figure 5:**
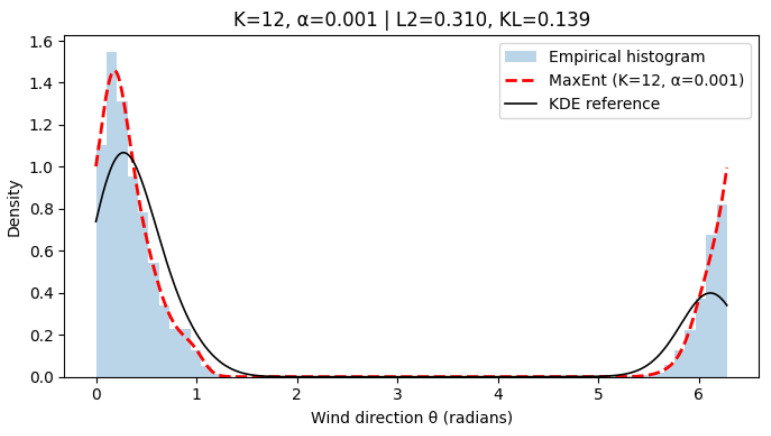
Reconstructed wind direction distribution using MaxEnt with K=12, $\alpha =10^{-3}$. Increasing model complexity allows for finer detail but may induce small oscillations near modal peaks.

Figure 3, Figure 4 and Figure 5 show representative results for increasing harmonic orders *K* and different regularization strengths α. With few harmonics (K=2), the method already captures the dominant wind regimes. Increasing *K* improves fidelity, while moderate regularization prevents oscillations. A very small α may introduce mild overfitting. Quantitative results in Table 2 confirm that intermediate values (K = 4–6, $\alpha =10^{-2}$) provide the best trade-off between accuracy and smoothness.

Overall, these experiments illustrate that the proposed continuous-domain formulation produces stable and interpretable density estimates while avoiding discretization or kernel bandwidth tuning.

## 6. Conclusions

This paper developed a continuous-domain formulation of the Maximum Entropy principle for multivariate density estimation under moment constraints. By combining convex duality with the theory of convex integral functionals, we showed that an infinite-dimensional entropy minimization problem over probability densities admits a finite-dimensional dual representation. This result provides a rigorous and tractable foundation for Maximum Entropy estimation without discretizing the underlying domain.

The introduction of relaxed moment constraints through convex potential functions allows for a unified treatment of exact constraints, box constraints, and quadratic relaxations. On the dual side, this leads to concave optimization problems whose analytical and numerical properties depend explicitly on the chosen relaxation while remaining amenable to standard optimization algorithms.

From a conceptual standpoint, the proposed framework bridges the gap between classical discrete Maximum Entropy formulations and genuinely nonparametric continuous models. It preserves the interpretability and structural simplicity of the entropy principle while extending its applicability to settings where discretization may introduce bias or instability.

The selection of appropriate feature functions remains a central issue in continuous-domain Maximum Entropy modeling and deserves further investigation.

Future work will focus on principled choices of feature families and on extensions to structured domains, including spatial, physically informed, or space–time settings with partial observations.

## Figures and Tables

**Table 1:** Meteorological observation attributes used in the wind direction reconstruction.

Feature	Unit	Description	Example
Timestamp	Unix	Observation timestamp	1,514,784,601
Flight ID	ICAO	Unique aircraft identifier	A8B75F
Latitude	Degree	Geographic latitude	52.43611
Longitude	Degree	Geographic longitude	3.61474
Altitude	Feet	Aircraft altitude	12,875
Wind component $w_{x}$	m/s	Eastward wind component	18.97
Wind component $w_{y}$	m/s	Northward wind component	6.64
Temperature	Kelvin	Ambient air temperature	250.85

**Table 2:** Quantitative comparison between MaxEnt and KDE on wind direction data.

*K*	α	Log-Likelihood	$L^{2}$ Distance	KL Divergence
2	0.01	−0.313	0.249	0.097
4	0.01	−0.312	0.299	0.126
6	0.01	−0.309	0.309	0.125
8	0.01	−0.305	0.308	0.131
12	0.001	−0.301	0.316	0.139

## Data Availability

The data analyzed in this study are publicly available on Figshare at https://doi.org/10.6084/m9.figshare.6970403.v1 [19].
